# Water and Air as Nourishment

**Published:** 1880-08

**Authors:** 


Water and Air as Nourishment.—The exploit of fasting forty days and nights, attempted by a mono-maniac named Tanner, in New York, will have terminated disastrously or otherwise ere this issue reaches all of its readers, and it will probably be gazetted to the world very soon thereafter, with all the details and particulars connected with the sacrilegious and unnecessary display of fool-hardy, if not brute endurance. The advantages to science that have been put forth by the aiders and abettors of this unnecessary exposure of the life of a lunatic, must be exceedingly few and small, if indeed, any worth recording should occur at all; while the attempt to emulate the example of deprivation and endurance, by the world's Redeemer, and to verify its truth or falsity, is sacrilege of the most infamous and revolting character to the mind of every Christian. But suppose he should continue to survive the forty days, he will still be *eighteen days* short of what was accomplished by a religious enthusiast, who practiced total abstinence from food for an interval of *fifty-eight days*. This man, Fergus Purden, died at his residence, No. 88 St. Mary street, in this city, Baltimore, on the 17th day of May, 1876, after a *total* abstinence from food for a period of 58 days.

It has been claimed that much practical information will be gained from Tanner's fasting, that may be turned to advantage in the treatment of inflammatory trouble of the mucous surface of the alimentary apparatus; as no food need be administered in such cases for a longer period without detriment or injury, than has been thought advisable heretofore. Ergo, nature and remedies could thus have better opportunity for restoring the healthy condition of parts or surfaces than usually occurs. Intelligent physicians have long been aware of the necessity for witholding all food except the blandest and most easily assimilable ; and even these articles are inhibited very often for days, in certain inflammatory and other conditions of the stomach and intestines, not only *without* injury but with positive good result, in the management of such cases. Were it not that nature *often* demands, in fact, *generally* calls for abstinence from food, more or less complete in disease, our "confrères of the high attenuations and little pellets," would be compelled to hang their golden harps upon the willows and soon find their occupation gone. Indeed, about the only *sane* thing the great expounder of the doctrine of homœopathy left behind him, was the inhibition of *feeding* the sick, and certainly his *true* followers are indebted for *all* the success that attends their cases, to the recuperative powers of nature, and abstinence from food. But Tanner is an *eclectic* and *spiritualist*, and belongs neither to the regular profession nor to homœopathy, and is therefore heterodox in physic and theology ; as such, it is not likely he will succeed in the enlightenment of any outside *his* own confrères and friends ; unless it should be to prove to the inhabitants of Gotham, that the microcosms of animal and vegetable matter in their croton water, are sufficiently sustaining to human life to warrant a reduction of their market money ; if, indeed, the faster should get his food from *no other* source or quarter. But *nous verrons.*

